# Incidence of Hospitalization for Respiratory Syncytial Virus Infection amongst Children in Ontario, Canada: A Population-Based Study Using Validated Health Administrative Data

**DOI:** 10.1371/journal.pone.0150416

**Published:** 2016-03-09

**Authors:** Andrea Pisesky, Eric I. Benchimol, Coralie A. Wong, Charles Hui, Megan Crowe, Marc-Andre Belair, Supichaya Pojsupap, Tim Karnauchow, Katie O'Hearn, Abdool S. Yasseen, James D. McNally

**Affiliations:** 1 Department of Pediatrics, Children’s Hospital of Eastern Ontario, University of Ottawa, Ottawa, Ontario, Canada; 2 Children’s Hospital of Eastern Ontario Research Institute, University of Ottawa, Ottawa, Ontario, Canada; 3 School of Epidemiology, Public Health and Preventive Medicine, University of Ottawa, Ottawa, Ontario, Canada; 4 Institute for Clinical Evaluative Sciences (ICES uOttawa), Ottawa, Ontario, Canada; Kliniken der Stadt Köln gGmbH, GERMANY

## Abstract

**Importance:**

RSV is a common illness among young children that causes significant morbidity and health care costs.

**Objective:**

Routinely collected health administrative data can be used to track disease incidence, explore risk factors and conduct health services research. Due to potential for misclassification bias, the accuracy of data-elements should be validated prior to use. The objectives of this study were to validate an algorithm to accurately identify pediatric cases of hospitalized respiratory syncytial virus (RSV) from within Ontario’s health administrative data, estimate annual incidence of hospitalization due to RSV and report the prevalence of major risk factors within hospitalized patients.

**Study Design and Setting:**

A retrospective chart review was performed to establish a reference-standard cohort of children from the Ottawa region admitted to the Children’s Hospital of Eastern Ontario (CHEO) for RSV-related disease in 2010 and 2011. Chart review data was linked to Ontario’s administrative data and used to evaluate the diagnostic accuracy of algorithms of RSV-related ICD-10 codes within provincial hospitalization and emergency department databases. Age- and sex-standardized incidence was calculated over time, with trends in incidence assessed using Poisson regression.

**Results:**

From a total of 1411 admissions, chart review identified 327 children hospitalized for laboratory confirmed RSV-related disease. Following linkage to administrative data and restriction to first admissions, there were 289 RSV patients in the reference-standard cohort. The best algorithm, based on hospitalization data, resulted in sensitivity 97.9% (95%CI: 95.5–99.2%), specificity 99.6% (95%CI: 98.2–99.8%), PPV 96.9% (95%CI: 94.2–98.6%), NPV 99.4% (95%CI: 99.4–99.9%). Incidence of hospitalized RSV in Ontario from 2005–2012 was 10.2 per 1000 children under 1 year and 4.8 per 1000 children aged 1 to 3 years. During the surveillance period, there was no identifiable increasing or decreasing linear trend in the incidence of hospitalized RSV, hospital length of stay and PICU admission rates. Among the Ontario RSV cohort, 16.3% had one or more major risk factors, with a decreasing trend observed over time.

**Conclusion:**

Children hospitalized for RSV-related disease can be accurately identified within population-based health administrative data. RSV is a major public health concern and incidence has not changed over time, suggesting a lack of progress in prevention.

## Introduction

Acute lower respiratory tract infections (ALRI) are the leading cause of morbidity and mortality in children. Of the pathogens responsible for ALRI, Respiratory Syncytial Virus (RSV) accounts for approximately 20% of pneumonia and 85% of bronchiolitis [1, 2]. RSV is a ubiquitous enveloped RNA paramyxovirus that infects nearly 100% of children within three years of birth [3, 4]. Approximately 30% of children develop clinical symptoms and 10% require medical attention, with 1 to 3% requiring hospitalization [5, 6]. Attempts to create a vaccine with beneficial adaptive immunity have been unsuccessful. The personal and healthcare costs associated with the acute illness and the potential long-term morbidity make RSV a major public health concern [7–9].

Due to its prominent role in pediatric healthcare, RSV surveillance and research, including developing predictive models and considering new interventions, remains a priority [10]. Routinely collected health administrative data has been proposed as a powerful way to conduct disease surveillance and health services research [11–13]. Although attractive, the value of the data is highly dependent on the availability and accuracy of codes. Without validation, the risk of misclassification bias is significant and study results may be difficult to interpret [14]. The accuracy of diagnostic codes used for identification of hospitalized RSV from within administrative data has not previously been determined.

The primary objective of this study was to validate an algorithm to identify children hospitalized for RSV infection from within Ontario’s population-based health administrative data. Secondary objectives were to determine the annual incidence of hospitalized RSV disease in Ontario and report the frequency of major risk factors within the RSV cohort.

## Methods

### Ethical issues

This study was approved by the Research Ethics Board of the Children’s Hospital of Eastern Ontario (CHEO) and by The Ottawa Hospital Research Institute (OHRI). This study complied with privacy regulations of the Institute for Clinical and Evaluative Science (ICES). To protect privacy, all cell sizes fewer than six individuals were suppressed and reported as n < 6. Consent was not obtained for participants for the use of their data in this study. All patient information was anonymized and de-identified prior to analysis.

### Data sources

This study used health administrative data from Ontario, Canada, a province with a population of 12.9 million residents in 2011 [15]. Within the universal healthcare coverage system, Ontario’s health administrative data captures data for all legal residents, accounting for >99% of the population. The Institute for Clinical Evaluative Sciences (ICES) maintains Ontario’s administrative databases through a comprehensive data-sharing agreement with the Ontario Ministry of Health and Long Term Care. Individual-level data are linked across databases using a unique, encrypted identification number based on the health card number. Identification of eligible children and those admitted to hospital during the study period was performed using the Canadian Institute of Health Information Discharge Abstract Data (CIHI-DAD), physician billings from the Ontario Health Insurance Health Plan (OHIP), the National Ambulatory Care Reporting System (NACRS) and Registered Persons Database and census area profiles (1996, 2001, 2006 Canadian Censuses) [16, 17].

To establish the true-positive reference cohort we first identified children under the age of 3 years residing within the Ottawa region who were potentially hospitalized for RSV at the Children’s Hospital of Eastern Ontario (CHEO) between January 1, 2010 and December 31, 2011. CHEO is the sole pediatric hospital in the Census Metropolitan Area (CMA) of Ottawa, with no other hospitals providing inpatient pediatric care. Two strategies were used to identify these potential cases: 1) Hospital Decision Support provided a list of all admissions from 2010 and 2011 that had an ICD-10 code relating to respiratory pathology or apnea (Appendix A in S1 File), 2) the Ottawa Regional Virology Laboratory, located at CHEO, identified patients who had respiratory virus testing during the study period. All children from the CMA of Ottawa admitted to CHEO had their testing completed by the Regional Virology Laboratory (confirmed by our chart review). During the study period the Regional Virology Laboratory relied on direct fluorescent antibody (DFA) staining followed by cell culture, if DFA negative, for the routine detection of RSV in nasopharyngeal aspirates and swabs. Based on internal laboratory data, DFA sensitivity and specificity versus cell culture and real-time PCR are 100% and 88% and 95% and 100%, respectively. From the two sources, admissions were eligible if the patient had a valid Ontario health card, resided in Ottawa at the time of admission (Appendix B in S1 File), and were less than 3 years of age. Children admitted to the Neonatal Intensive Care Unit (NICU) immediately following birth were excluded because RSV does not cause symptoms until children are exposed after birth. The child was considered a potential case if admitted to hospital within 30 days of a positive virology test for RSV. Charts of all potential cases were reviewed. Admissions were classified as true-positive if the child tested positive for RSV within 72 hours of admission and if the signs and symptoms responsible for hospital admission were consistent with RSV pathophysiology. Admissions were coded as index or recurrent and classified according to their disease-type based on pre-defined diagnostic criteria for pneumonia, apnea, bronchiolitis and upper respiratory tract infection (Appendix C in S1 File) Chart data was extracted directly into a case-report form developed using REDcap (Research Electronic Data Capture), a secure web-based application designed for building and managing online surveys and databases [18]. Two chart abstractors were trained by one investigator (AP), who ensured consistency through a quality assurance review of 20% of charts. Following training, the agreement between reviewers for RSV-status and disease-type classification was 100%.

The true-negative reference cohort for the study represented all children not in the true-positive cohort who were admitted to CHEO in 2010 or 2011, were under 3 years and resided in the Ottawa CMA. The Registered Persons Database was used to identify all children with a valid health card that resided in Ottawa and were under the age of three in 2010–2011. From this group we determined those who were admitted to CHEO during the study period based on facility code.

### Statistical analysis: validation of the RSV algorithm

The true-positive and true-negative reference standard cohorts were used to validate the accuracy of the algorithms selected for testing (*a priori)* within the hospitalization (CIHI-DAD) and emergency department (NACRS) databases. As this study only included children born after 2002, the algorithm considered only the International Classification of Diseases, 10^th^ revision (ICD-10) diagnostic codes. The algorithm included one or more of the following ICD-10 codes: J12.1, J20.5, J21.0, and B97.4 (Table 1). A pre-specified secondary analysis was performed to determine whether specific clinical presentations could be identified by individual ICD-10 codes (i.e. patients with pneumonia were assigned the RSV pneumonia ICD-10 code). During the chart review information was also collected on pediatric intensive care unit (PICU) admission, non-invasive ventilation, intubation and length of stay (LOS).

**Table 1:** International Classification of Disease 10 codes for patient diagnoses and characteristics.

Description	Code type	Code
Any Hospitalized RSV	ICD10	J12.1, J20.5, J21.0, and B97.4
Pneumonia	ICD10	J12.1
Apnea	ICD10	J12.1, J21.0, P28.4
Bronchiolitis	ICD10	J12.1, J21.0, P28.4, P28.8, R06.8
Upper Respiratory Tract Infection	ICD10	B974 plus one of: J00, J01.0-J01.9, J02.8, J02.9, J03.0-J03.8, J04.0-J04.2. J05.0, J05.1. J06.9
CHD	ICD10	Q20.0-Q20.9, Q21.0-Q21.9, Q22.0-Q22.9, Q23.0- Q23.9, Q24.0- Q24.9, Q25.0- Q25.9, Q26.0-Q26.9
Cardiopulmonary bypass	Intervention code	1LZ37LAGB
Trisomy 21	ICD10	Q90.0, Q90.1, Q90.2, Q90.9
BPD	ICD10	P27.1, P27.8, P27.9
PICU Admission	Special Care Unit (from CIHI-DAD)	50 = NICNU; 51 = NICNU, Level 1 (new in 2008); 52 = NICNU, Level 2 (new in 2008); 53 = NICNU, Level 3 (new in 2008); 70 = PICU
Intubation	Intervention	1GZ31CAND
Non-invasive ventilation	Intervention	1GZ31CBND, 1GZ31CBEP, 1GZ31CAMP, 1GZ31JAMD, 1GZ31JANC, 1GZ31JAPK
Hospital Length of Stay	CIHI-DAD	LOS
PICU length of stay	CIHI-DAD (Special care unit)	LOS for 70 = Pediatric Intensive Care Nursing Unit

Abbreviations: BPD = Bronchopulmonary Dysplasia; PICU = Pediatric Intensive Care Unit; RSV = Respiratory Syncytial Virus; LOS = Length of Stay; NICNU = Neonatal Intensive Care Nursing Unit

### Study design: incidence and risk factors

Using the validated algorithm, we determined the annual incidence hospitalized RSV infection from fiscal year (FY, April 1 to March 31) 2005 to 2013. Crude and age- and sex-standardized incidence rates were calculated per 1000 children for three age groups <1 year, <3 years, 1–3 years) based on census and inter-censal estimates of baseline population characteristics [16]. ICD-10 and procedural codes were used to report descriptive statistics and compare risk factors prevalence.

### Statistical analysis and sample size calculation

The accuracy of each code and algorithm was evaluated using sensitivity, specificity, positive predictive value (PPV) and negative predictive value (NPV) parameters. For each, we calculated 95% confidence intervals using the binomial distribution. With anticipated estimates in excess of 80%, it was calculated that approximately 300 RSV cases would be required to generate confidence interval error margins under 5%. We anticipated identifying 150 hospitalized RSV cases at CHEO per year, with 75% of those being eligible for the validation study.

All data manipulation and analyses were conducted by an ICES analyst (CAW, MAB) under the supervision of the investigators (JDM, EIB, AP) using SAS Version 9.3 (SAS Institute Inc., Cary, NC, USA). Confidence intervals for the incidence rates were calculated using the gamma distribution. A two-sample t-test was used to assess differences in means, and chi-square test of association was used to compare the true-negative and true-positive cohorts for continuous and categorical variables, respectively. We used ordinary least squares (OLS) regression to evaluate the average increase in the standardized incidence rate and the linear trend in the risk factors over time.

## Results

### Reference standard cohort

**Fig 1:**
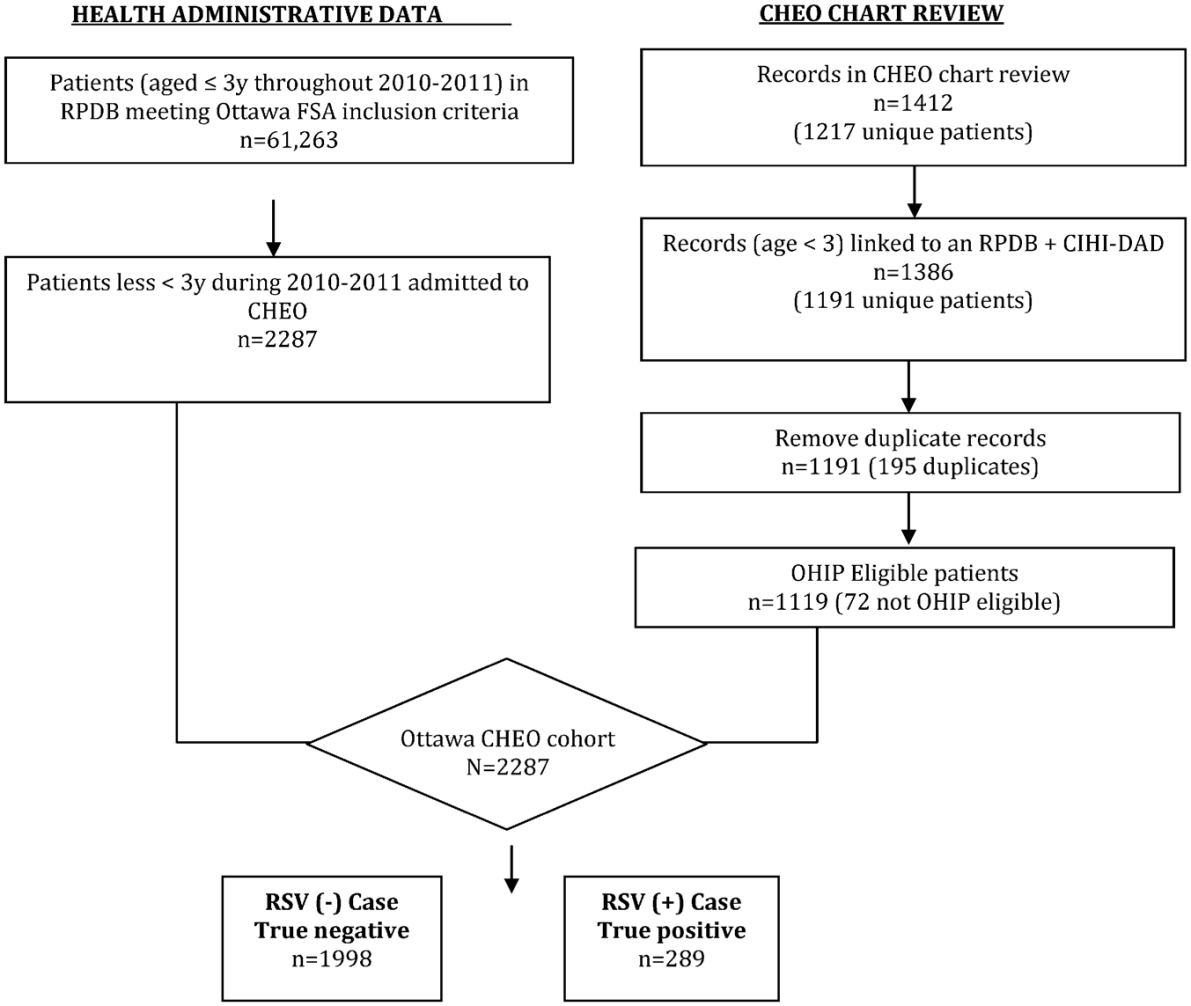
Flow diagram of reference standard cohort creation against which algorithms were validated. Abbreviations: CHEO = Children’s Hospital of Eastern Ontario; CIHI-DAD = Canadian Institute of Health Information—Discharge Abstract Database; FSA = Forward Sortation Area; OHIP = Ontario Health Insurance Plan; RPDB = Registered Person Database; RSV = Respiratory Syncytial Virus.

A total of 1411 CHEO hospital admissions were identified as potential RSV cases using the two search strategies (Fig 1). Chart review determined 327 were true-positive cases with the most responsible diagnosis as follows: bronchiolitis (n = 298), pneumonia (n = 19), apnea (n<6) and upper respiratory tract symptoms (n<6). There were <6 cases of nosocomial RSV and 10 cases where the patient had a positive DFA but was admitted for symptomatology unrelated to RSV.

Of the 1411 potential cases, 1386 (98%) were successfully linked at ICES. Removing admissions that were not OHIP-eligible during the study period (n = 72) or represented second hospital admission (n = 195) reduced the final number of potential cases to 1119, with 289 true-positive cases. The Registered Persons Database and CIHI-DAD identified 2287 children under the age of 3 years from the Ottawa region who had OHIP eligibility and were admitted to CHEO during the two-year study period, producing a true-negative cohort size of 1998.

### Algorithm Validation

The accuracy of the proposed algorithm (any of the following: J12.1, J20.5, J21.0, B97.4) for classifying Ottawa children according to their diagnosis for the CIHI-DAD is presented in Table 2. Included among the true-negative cohort were 73 children with respiratory admission symptoms, but for whom there was no respiratory virus testing: none (0%) were identified by the algorithm as being an RSV case. When the full RSV algorithm was evaluated against the NACRS database the results were as follows: sensitivity 49.5% (95%CI: 43.7–55.3%), specificity 99.6% (95%CI: 99.5–99.8%), PPV 94.1% (95%CI: 90.3–97.8%) and NPV 93.2% (95%CI:92.1–94.2%).

**Table 2:** Algorithm validation results of full RSV-positive cohort and specific disease pathophysiology against the Canadian Institute of Health Information—Discharge Abstract Database.

N = 2287	Hospitalized RSV*	Bronchiolitis	Apnea	Pneumonia	URTI
Sensitivity (95% CI)	97.9 (95.5, 99.2)	94.6 (91.3, 97.0)	44.4 (13.7, 78.8)	34.0 (21.2, 48.8)	14.3 (1.8, 42.8)
Specificity (95% CI)	99.6 (98.2, 99.8)	99.3 (98.8, 99.6)	100 (99.8, 100)	99.6 (99.2, 99.8)	100 (99.8, 100)
Positive predictive value (95% CI)	96.9 (94.2, 98.6)	94.6 (91.3, 97.0)	80.0 (28.4, 99.5)	63.0 (42.4, 80.6)	100 (15.8, 100)
Negative predictive value (95% CI)	99.4 (99.4, 99.9)	99.3 (98.8, 99.6)	99.8 (99.5, 99.9)	98.5 (98.0, 99.0)	99.5 (99.1, 99.7)

Abbreviations: CI = Confidence Interval; RSV = Respiratory Syncytial Virus; URTI = Upper Respiratory Tract Infection.

*Full algorithm used for “Hospitalized RSV” was any of the following: J12.1, J20.5, J21.0, and B97.4.

**Exact 95% CIs were calculated using the binomial distribution.

### Algorithm Validation—Sensitivity and Subgroup Analyses

In addition to evaluating the algorithm performance during the entire calendar year, as above, the algorithm performance was also evaluated outside of RSV season, specifically from April 1 to October 31. Of the 19 hospitalized RSV cases occurring outside of the RSV season, 18 were correctly identified by the algorithm (94.7%). Algorithm performance was also compared in children above and below 6 months of age: all four accuracy parameters remained above 95% and differed by less than 2% between groups (Table A in S1 File).

The ability of individual ICD-10 codes to identify the presence of specific RSV pathology (i.e. apnea, pneumonia) was evaluated (Table 2). Chart review data was also used to evaluate the accuracy of health administrative codes for LOS, PICU admission, endotracheal intubation and non-invasive ventilation (Table C in S1 File). There was 100% agreement for length of stay; and the PPV and sensitivity for PICU admission and intubation both exceeded 90%. For non-invasive ventilation, although the PPV was high (87.5%) the sensitivity for the combination of codes was only 28% (95%CI:12–49%).

### Cohort Creation

**Fig 2:**
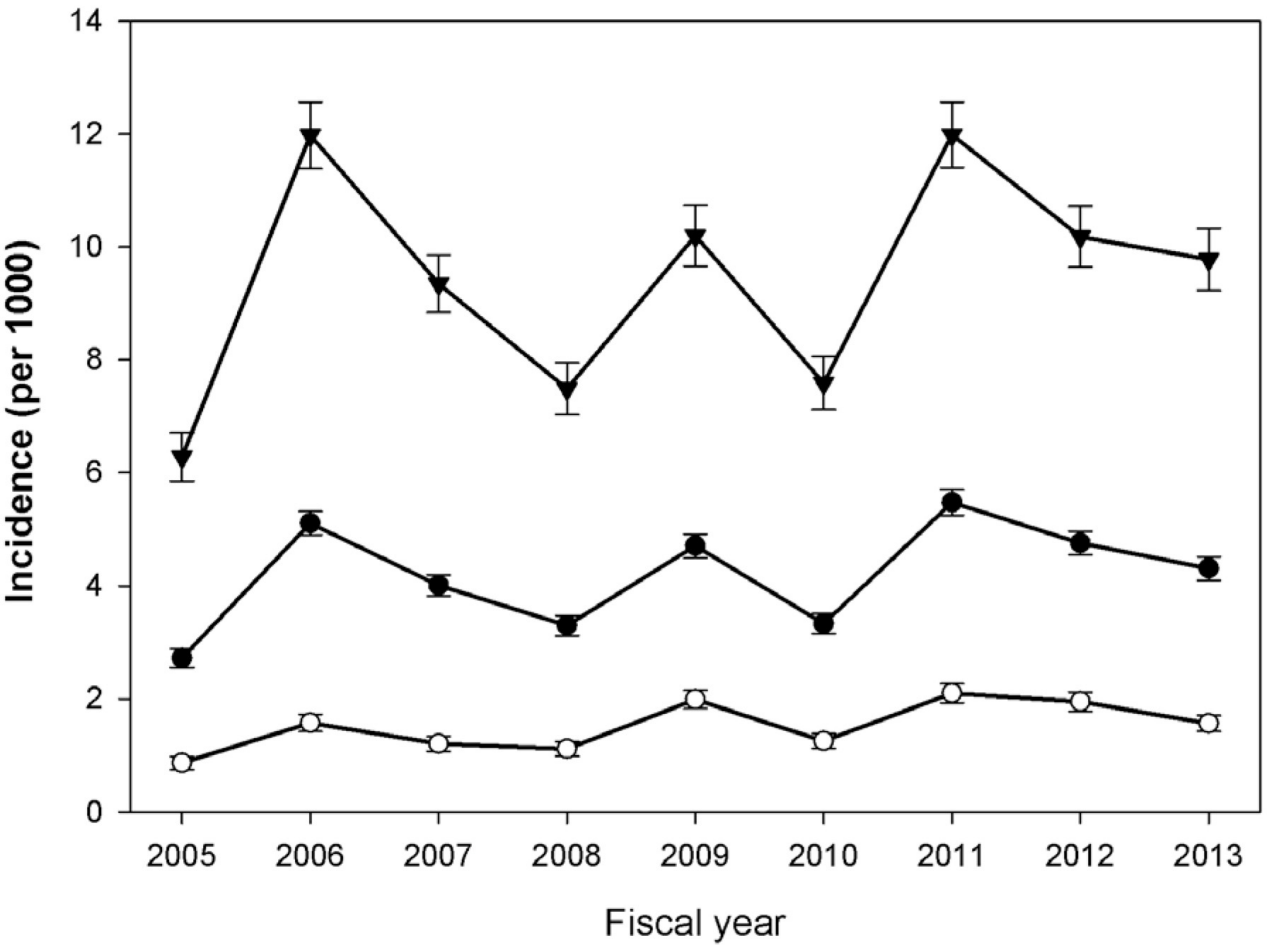
Incidence of hospitalized RSV (per 1000 children) 2005–2013 with 95% confidence intervals. Legend: Incidence per 1000 person years is given for each fiscal year from 2005 to 2013. Error bars shown are the calculated 95% confidence intervals. Closed circles (•) represent the incidence in the full Ontario cohort of children under 3 years of age. Closed triangles (▾) represent the incidence for children under 1 year of age. Open circles (ο) represent the incidence for children between 1 and 3 years of age.

The validated algorithm for Hospitalized RSV, using CIHI-DAD codes (Table 1), was applied to the full provincial health administrative data to develop the Ontario RSV cohort. It identified 19815 children under 3 years of age hospitalized for RSV between April 1, 2005 and March 31, 2013. Of these, 11449 (57.8%) were male and the median age at hospitalization was 4 months (IQR: 2, 10). RSV accounted for 8.8% of admissions for children under 1 year, 4.5% between 1 and 2 years and 2.7% for those between 2 and 3 years (Table B in S1 File). The sex-standardized incidence (per 1000 person years) was 4.8 (CI: 4.72–4.85) and 10.2 (CI: 10.03–10.35) for patients under 3 years and patients under 1 year of age, respectively (Table D in S1 File). Sex-stratified incidence by fiscal year was calculated (Fig 2), and no significant increasing or decreasing linear trend was observed over the study period (Beta 0.21, 95% CI -0.39 to 0.83, P = 0.42). Year-to-year variability in sex-standardized incidence was evident, ranging from as low as 6.3 (CI: 5.9, 6.7) in 2005 to as high as 12.0 (CI: 11.4, 12.6) per 1000 in 2011.

### Risk factors, Disease Severity and Length of Stay

The prevalence of major RSV risk factors was determined in the Ontario RSV cohort and compared to Ontario children not hospitalized with RSV (Table 3). The percentage of children born less than 33 weeks (3.7% vs. 1.4%) or between 33 and 36 weeks (11.3% vs. 6.4%) was significantly higher (p = 0.0001) within the Ontario RSV cohort, when compared to children not hospitalized for RSV. Similarly, when compared to children not hospitalized for RSV, more children in the Ontario RSV cohort had CHD requiring surgery (1.0% vs. 0.2%, p = 0.0001), Trisomy 21 (0.7% vs. 0.1%, p = 0.0001) and chronic lung disease arising the neonatal period (1.4% vs. 0.2%, p = 0.0001). Over the study period, 16.7% of children under the age of 3 who were hospitalized with RSV had a major risk factor; considering any CHD, with or without surgery, as a risk factor only increased the percentage to 19.2%. Evaluation of the linear trend determined that the proportion of children with at least one major risk factor has decreased significantly over the study period (Beta 0.29%, 95% CI -0.53 to -0.05, p = 0.02) (Figure A in S1 File).

**Table 3:** Prevalence of major RSV risk factors in Ontario children less than 3 years.

Risk Factor	Risk Factor Details	All	Hospitalized RSV N = 19,815	Controls	p-value
		N = 1,615,353		N = 1,595,538	
**BPD**	ICD-10 codes: ‘P27.1’	2482 (0.2%)	187 (0.9%)	2295 (0.1%)	<0.0001
	ICD 10 codes: ‘P27.1, P27.8, P27.9’	4094 (0.3%)	273 (1.4%)	3821 (0.2%)	<0.0001
**Trisomy 21**	NA	2072 (0.1%)	145 (0.7%)	1927 (0.1%)	<0.0001
**CHD**	NA	25,109 (1.6%)	1,263 (6.4%)	23,846 (1.5%)	<0.0001
**CHD + surgery**	No. of surgeries	3,821	245**	3,576	<0.0001
	No. of patients	3,267 (0.2%)	199 (1.0%)	3,068 (0.2%)	<0.0001
**Gestational age**	Missing	8,809 (0.5%)	656 (3.3%)	8,253 (0.5%)	<0.0001
	<33 weeks	22,362 (1.4%)	742 (3.7%)	21,620 (1.4%)	<0.0001
	33–36 weeks	103,042 (6.4%)	2227 (11.2%)	100,815 (6.3%)	<0.0001
	>36 weeks	1,481,040 (91.7%)	16,190 (81.7%)	1,464,850 (91.8%)	<0.0001
**Patients** **without any** **of the four risk factors**	CHD, *Prematurity, Trisomy 21, BPD	1,465,803 (91.3%)	15,482 (80.0%)	1,450,321 (91.4%)	<0.0001
	CHD+Surgery, *Prematurity, Trisomy 21, BPD	1,477,103 (92.0%)	15,957 (83.3%)	1,461,146 (92.1%)	<0.0001
**Patients** **with at lease one** **of the four risk factors**	CHD, *Prematurity, Trisomy 21, BPD	140,641 (8.7%)	3,677 (19.2%)	136,964 (8.6%)	<0.0001
	CHD+Surgery, *Prematurity, Trisomy 21, BPD	129,341 (8.0%)	3,202 (16.7%)	126,139 (7.9%)	<0.0001

Abbreviations: BPD = Bronchopulmonary Dysplasia; CHD = Congenital Heart Disease; RSV = Respiratory Syncytial Virus

** Surgeries before RSV = 119; Surgeries after RSV = 126.

* Prematurity defined as less than 37+0 weeks gestational age

Median hospital LOS was 3 days (IQR: 2, 5). Of the hospitalized cohort, 5.6% (95% CI 5.2% to 5.9%) were admitted to PICU and 3.1% (95% CI 2.9% to 3.3%) were intubated. There was no significant change for hospital LOS, likelihood of PICU admission or endotracheal intubation evident over the study period (Figure B in S1 File). Children admitted to PICU and requiring intubation had a median LOS of 11 days (IQR: 8,18), while those not intubated had a median LOS of 6 days (IQR: 4,9).

## Discussion

In this study we validated an algorithm of ICD-10 diagnostic codes to be both highly sensitive and specific for the identification of hospitalized-RSV within Ontario health administrative data. Using the algorithm we reported the annual incidence of RSV from 2005 to 2013, finding no change over time. Finally, we determined that fewer than 20% of children admitted for RSV had one or more major risk factors.

Health administrative data have recently been recognized as a potentially fruitful resource for high-quality, population-based disease surveillance and health resource utilization research [19, 20]. Recognizing this potential, a number of researchers have published RSV-related studies using health administrative data [11–13]. To our knowledge this study is the first to use a validated algorithm to study RSV at a population level using administrative data. We used primary chart data to test the accuracy of an ICD-10 based algorithm within hospitalization and emergency department databases. The algorithm performed extremely well within the hospitalization database with sensitivity, specificity, PPV and NPV all exceeding 96%. These findings strongly suggest that, under proper conditions, an algorithm of ICD-10 codes can accurately identify cases of hospitalized RSV. The lack of accuracy within the emergency department database affirms that not all databases are equivalent and reinforces that each requires assessment before utilization [14]. Despite the limited number of validation studies in pediatrics [21], there have been a few specifically targeting respiratory illnesses that report similar algorithm performance. For example, in a study evaluating pediatric asthma within the Ontario health administrative data, To *et al*. determined that an algorithm of physician visits and hospitalization had 89% sensitivity and 72% specificity [22]. Further, within the Danish National Patient Registry, excellent code accuracy was observed for hospitalized asthma (sensitivity 99%, PPV 85%) [23]. Finally, within the Pediatric Health Information System, an algorithm of pneumonia codes had sensitivity and specificity above 80% for community acquired pneumonia in children without chronic disease [24]. However, not all pediatric validation studies have returned such positive results, as demonstrated in studies for rotavirus infections and Kawasaki Disease [25, 26]. Despite the excellent accuracy of our algorithm when applied to Ontario health administrative data, we caution against its use in other jurisdictions without prior validation [27].

We confirmed that children under one year are significantly more likely to be hospitalized for RSV than those between one and three [13]. In infants, we calculated an annual incidence of 10 per 1000 and reported that RSV was responsible for 9% of hospital admissions. This confirms RSV as a major Canadian public health issue. Further, our observation that incidence has been constant does not support older studies suggesting a rise in hospitalization rate [8, 28] and is consistent with a recent US study [13]. Our calculated RSV incidence fits well with literature and rates from large high-quality prospective cohort studies. For example, our Ontario incidence is comparable to that reported by Hall *et al*. (11 per 1000) who followed a large cohort of children and applied a case-definition that required both virology confirmation and clinical data [2]. However, our Ontario rate was notably lower when compared with studies using health administrative data from England (24 per 1000) [11], Spain (41 per 1000) [12] and the United States (26 per 1000) [13]. Differences could relate to variability in population susceptibility, ambulatory treatment approaches and thresholds for admission. For example, the average length of hospital stay in a UK study was one day, suggesting admission of less unwell children [11]. Furthermore, differences could also relate to variability in study methodology, specifically case-identification and mathematical assumptions. The aforementioned HAD studies use unvalidated algorithms that valued sensitivity at the expense of specificity [11]. Murray et al, for example, considered any bronchiolitis-related ICD-10 code as an RSV-positive case and did not require virology confirmation. The inclusive nature of this application of ICD-10 codes to identify cases and the assumption that all cases of bronchiolitis were secondary to RSV likely overestimates the incidence of RSV-related disease [1,2]. These identified differences in incidence rates underscore the importance of using a validated algorithms for HAD research.

To enrich our understanding of the hospitalized RSV cohort, we evaluated variables reflective of disease severity, patient characteristics, specifically the presence of major RSV risk factors, and variations in health resource utilization over time. We observed no change in average hospital LOS, PICU admission rate or endotracheal intubation rate. This suggests that there has been no significant improvement in either the prevention of severe RSV or the treatment of hospitalized RSV in the general population over the past decade. Further, risk factor evaluation identified that less than 20% of children hospitalized for RSV had at least one major risk factor for RSV. Similar to programs in other countries, the Ontario RSV prophylaxis program seeks to reduce RSV-related morbidity and mortality through the monthly administration of an RSV antibody (pavilizumab) to children considered high risk because of the presence of at least one major RSV risk factors, which currently include the following: prematurity, congenital heart disease, Trisomy 21 and Chronic Lung Disease arising the neonatal period. The observation that the proportion of hospitalized cases with a major risk factor declined over the study period implies improved effectiveness of the Ontario RSV prophylaxis program for the subset of children considered high-risk [29]. Finally, significant year-to year variability in RSV incidence rate was evident across the study period, consistent with previous studies [2, 30]. This significant year-to-year variability is relevant as it strongly supports evaluating new interventions and policies as part of controlled trials, otherwise multiple years of surveillance would be required before any conclusions could be drawn. Altogether our study findings suggest that novel research and policy changes will be required to further reduce the burden of RSV in Canada.

Our study has a number of limitations. Firstly, the reference-standard cohort was identified retrospectively and our search strategy relied upon accurate documentation in health records. Second, during the study period the Regional Virology Laboratory relied on RSV-specific DFA coupled with cell culture, which may have missed some cases of RSV-positive bronchiolitis. Third, because the validation study was performed at a single institution, we cannot be certain that our algorithm will have identical accuracy elsewhere. Based on the findings of multi-center validation studies on pneumonia and asthma and on the fact that Ontario uses coders trained uniformly by CIHI, the algorithm performance is likely consistent across Ontario hospitals [23, 24]. Further, our analysis does not consider that some children received pavilizumab, which would have reduced both the incidence and strength of association with major risk factors. Finally, it is important to note that both the algorithm and calculated incidence rates apply only to hospitalized RSV; less severe RSV disease, such as visits to the emergency department, is outside of the scope of this study. The strengths of our study include the strict case-definition that includes virology confirmation, the use of a validated algorithm to identify the RSV cohort and the large population-based cohort used for evaluation.

## Conclusion

We validated an algorithm to identify children hospitalized with RSV from within Ontario’s health administrative data and used it to estimate incidence and evaluate risk factor prevalence. Findings confirm RSV to be a significant burden to young children and the health care system. The availability of a validated algorithm will facilitate further cost-effective epidemiological, surveillance and health services research.

## Supporting Information

S1 File Appendix A: CHEO electronic health records search.
Appendix B: Postal codes considered within the Census Metropolitan Area of Ottawa.
Appendix C: Diagnostic criteria for specific pathophysiology.
Table A. Algorithm validation by age at hospitalization, against the Canadian Institute of Health Information—Discharge Abstract Database.
Table B. Validation of HRU codes.
Table C. RSV admissions stratified by age between 2005–2013.
Table D. Standardized RSV hospital admissions by sex and age between 2005–2013.
Figure A. Proportion of children hospitalized with RSV in whom major risk factors were present from 2005–2013.
Figure B. PICU admission and intubation rates for children hospitalized with RSV 2005–2013.
(DOCX)

S2 File Reporting Checklists. (DOCX)
