# Supplementary material for: Incidence of Hospitalization for Respiratory Syncytial Virus Infection amongst Children in Ontario, Canada: A Population-Based Study Using Validated Health Administrative Data
Source: PLoS One. 2016 Mar 9;11(3):e0150416. doi: 10.1371/journal.pone.0150416 (PMC4784925; doi:10.1371/journal.pone.0150416)
Supplement: S1 File — Appendix A: CHEO electronic health records search. Appendix B: Postal codes considered within the Census Metropolitan Area of Ottawa. Appendix C: Diagnostic criteria for specific pathophysiology. Table A. Algorithm validation by age at hospitalization, against the Canadian Institute of Health Information—Discharge Abstract Database. Table B. Validation of HRU codes. Table C. RSV admissions stratified by age between 2005–2013. Table D. Standardized RSV hospital admissions by sex and age between 2005–2013. Figure A. Proportion of children hospitalized with RSV in whom major risk factors were present from 2005–2013. Figure B. PICU admission and intubation rates for children hospitalized with RSV 2005–2013. (DOCX) [file pone.0150416.s001.docx]

**Supporting Information**

**S1 File**

Appendix A: CHEO electronic health records search.

Appendix B: Postal codes considered within the Census Metropolitan Area of Ottawa.

Appendix C: Diagnostic criteria for specific pathophysiology.

Table A. Algorithm validation by age at hospitalization, against the Canadian Institute of Health Information – Discharge Abstract Database.

Table B. Validation of HRU codes.

Table C. RSV admissions stratified by age between 2005-2013.

Table D. Standardized RSV hospital admissions by sex and age between 2005-2013.

Figure A. Proportion of children hospitalized with RSV in whom major risk factors were present from 2005-2013.

Figure B. PICU admission and intubation rates for children hospitalized with RSV 2005-2013.

**Appendix A: CHEO electronic health records search.**

**ICD-10 codes included for the electronic health records search:**

J00 to J22:

- J01 (all inclusive) – Acute sinusitis
- J00 – Acute nasopharyngitis
- J02 (J02.0 02.8 & J02.9) – Acute pharyngitis, due to other streptococcal, specified organism and unspecified
- J03 (J03.0 J03.8 & J03.9 – Acute tonsillitis, streptococcal, other specified organisms and unspecified
- J04 (J4.0, J4.1 & J04.2) – Acute laryngitis, Tracheitis & laryngotracheitis
- J05 (J05.0 & J05.9) – Croup & epiglotitis
- J06 (J06.0, J06.8 & J06.9) – Acute upper respiratory tract infections of multiple sites
- J09 (all inclusive) - Influenza
- J10 (J10.0, J10.1 & J10.8) – Influenza due to other identified influenza virus
- J11 (J11.0, J11.1 & J11.8) – Influenza virus not identified
- J12 (J12.0, J12.1, J12.2, J12.3, J12.8, J12.9) – Viral pneumonia not elsewhere classified
- J13 (all inclusive) – Pneumonia due to streptococcus
- J14 (all inclusive) – Pneumonia due to Hemophilus Influenza
- J15 (all inclusive) – Bacterial pneumonia not elsewhere classified
- J16 (all inclusive) – Pneumonia due to other infectious organism
- J17 (all inclusive) – Pneumonia in diseases classified elsewhere
- J18 (all invlusive) – Pneumonia, organism unspecified
- J20 (all inclusive) – Acute bronchitis
- J21 (all inclusive) – Acute bronchiolitis
- J22 (all inclusive) – Unspecified acute lower respiratory tract infection

J30.1 to J31.0:

- J31 (J31.0) – Chronic Rhinitis

J34.8:

- J38.80 – Mucositis of he nose and nasal sinuses
- J34.88 – Other specified disorders of nose and nasal sinuses

J36 – Peritonsillar abscess

J38.3 to 38.5 & 38.7:

- J38 (J38.3, J38.4 & J38.5) – other disease of the vocal cords, oedema of larynx & laryngeal spasm
- J38.7 – Other diseases of larynx

J39.0 to J39.2:

- J39 (J39.8 & J39.9) – Disease of the upper respiratory tract unspecified

J40. to J42:

- J40 (all inclusive) – Bronchitis bronchitis not specified as acute or chronic
- J41 (all inclusive) – Simple and mucopurulent chronic bronchitis
- J42 – Unspecified chronic bronchitis

J45.00 to J47.:

- J45 (all inclusive) – Asthma

J80. to J81.:

- J81 – Pulmonary oedema

J85.0 to J91.:

- J85 (all inclusive) – Abscess in lung and mediastinum
- J86 (all inclusive) – Pyothorax
- J90 – Pleural effusion
- J91 – Pleural effusion in condition classified elsewhere

J93.0 to J94.9:

- J93 (all inclusive) – Pneumothorax
- J94 (all inclusive) – other pleural conditions (chylous effusion, fibrothorax, haemothorax, other specified pleural conditions, pleural conditions unspecified)

J95.3 & J95.9

- J95.3 – Chronic pulmonary insufficiency following surgery
- J95.9 – Post -procedure respiratory disorder, unspecified

J96. To J99

- J96 (all inclusive) – Respiratory failure not elsewhere classified
- J98 (all inclusive) – Other respiratory disorders
- J99 (all inclusive) – Respiratory disorders in diseased classified elsewhere

B28.4 – apnea, neonate

P28.8 – acute life threatening event, neonate

R06.8 – other abnormality of breathing, older child (outside of neonatal period)

**Excluded ICD-10 J codes – patients with only these J codes were removed:**

J30.0, J31.1, J31.2, J32.0, J32.1, J32.2, J32.3, J32.4, J32.8, J32.9, J33.0, J33.1, J33.8, J33.9, J34.0, J34.1, J34.2, J34.80, J34.88, J35.0, J35.1, J35.2, J35.3, J35.8, J35.9, J37.0, J37.1, J38.0, J38.01, J38.02, J38.09, J38.1, J38.2, J38.6, J39.3, J43.0, J43.1, J43.2, J43.8, J43.9, J44.0, J44.1, J44.8, J44.9, J60, J61, J62.0, J62.8, J63.0, J63.1, J63.2, J63.3, J63.4, J63.5, J63.8, J64, J65, J66.0, J66.1, J66.2, J66.8J67.0, J67.1, J67.2, J67.3, J67.4, J67.5, J67.6, J67.7, J67.8, J67.90, J67.99, J68.0, J68.1, J68.1, J68.2, J68.3, J68.4, J68.6, J68.9, J69.0, J69.1, J69.8, J70.0, J70.1, J70.2, J70.3, J70.4, J70.8 J70.9, J82, J84.0, J84.1, J84.8, J84.9, J92.0, J92.9, J95.00, J95.01, J95.02, J95.08, J95.1, J95.2, J95.4, J95.5, J95.80, J95.81, J95.88, J95.9, J99.0, J99.1, J99.8, J141, J143, J144,

**Appendix B: Postal codes considered within the Census Metropolitan Area of Ottawa.**

K0A, K1A, K1B, K1C, K1E, K1G, K1H, K1J, K1K, K1L, K1M, K1N, K1P, K1R, K1S, K1T, K1V, K1W, K1Y, K1X, K1Z, K2A, K2B, K2C, K2E, K2G, K2H, K2J, K2K, K2L, K2M, K2P, K2R, K2S, K2T, K2V, K2W, K4A, K4B, K4C, K4M, K4P, K4K, K7C, K7S

**Appendix C: Diagnostic criteria for specific pathophysiology.**

**Pneumonia^3,6,7,10,12^**

Must meet criteria 1 and 2:

1. Radiographic evidence of parenchymal infection with one of the following present on the CXR report, reported by a pediatric radiologist at CHEO.
   - New or progressive infiltrate
     - if more than one CXRs are done, the infiltrate must be persistent
   - Consolidation
   - Cavitation
   - Pneumatoceles (if under 1 year of age)
2. At least three of the following:
   - Fever or hypothermia (with no other recognized cause)***
   - Leukopenia or leukocytosis
   - New onset of purulent sputum or change in character of sputum or increased respiratory secretions or increased suctioning
   - New onset of worsening cough or dyspnea, apnea or tachypnea*
   - Rales or bronchial breath sounds on auscultation
   - Worsening gas exchange
     - Persistent pulse oximeter < 92%
     - Increased oxygen requirement from baseline
     - Rise in ventilator demand or carbon dioxide (on a blood gas)

**URTI^1,10^**

Must meet criteria 1 and 2

1. Does not meet criteria for penumonia or bronchiolitis, as previously defined. Meets criteria A., B. AND C..
2. No consolidation or infiltrate on CXR reported by a CHEO pediatric radiologist
3. No evidence of worsening gas exchange:
   - 1. No persistent desaturation, defined as <92% requiring the application of oxygen
     2. In children with chronic oxygen requirements: no rise in oxygen requirements from baseline
     3. No elevation in carbon dioxide, as measured on a blood gas
     4. In children with chronic hypercapnia: no rise in carbon dioxide from baseline
4. No bilateral wheeze, rhonchi or crackles on physical examination
5. Two or more of the following present:
   - 1. Fever or hypothermia (hypothermia only if < 1 year)***
     2. Rhinorrhea or nasal congestion
     3. Erythema of pharynx or sore throat
     4. Cough
     5. Hoarseness
     6. Purulent exudate in throat
     7. If < 1 year: bradycardia or apnea

**Definitive Bronchiolitis^2,11,15^**

Must meet criteria 1 and 2, and 3 when applicable.

1. Evidence of prior or active upper respiratory tract involvement. 1 of the following must be documented as part of a historical prodrome or current finding on admission to hospital.

- Rhinorrhoea
- Nasal congestion

1. Evidence of lower respiratory tract dysfunction. Criteria A. AND B. must be met.
2. Tachypnea* or respiratory distress **
3. Widespread wheeze or crackles on auscultation
4. If a CXR was completed: there was no evidence consolidation, infiltrate or pneumatocele as reported by a CHEO pediatric radiologist

**Suspected Bronchiolitis^2,11,15^**

Must meet criteria 1 and 2 when applicable.

1. Evidence of lower respiratory tract dysfunction. Criteria A. and B. must be met.
   - 1. Tachypnea* or respiratory distress **
     2. Wheeze or crackles on auscultation (NB unclear if widespread)
2. If a CXR was completed: there was no evidence consolidation, infiltrate or pneumatocele as reported by a CHEO pediatric radiologist

**Apnea^2^**

*Definitive apnea*

One of the following is observed and documented by a health-care provider:

- Cessation in respiration for 20 seconds
- Bradycardia (HR <80) with associated cyanosis or oxygen saturation below 90%.

*Suspected apnea*

- History of a cessation of breathing with associated change in colour or muscle tone.

*Tachypnea

• Age 0–2 months: >60

• Age 2–12 months: >50

• Age 1–5 Years: >40

**Respiratory distress is defined as the presence of one of the following^18^:

- Dypsnea
- Retractions
- Grunting
- Nasal flare
- Cough
- O2 sats < 94% RA
- Apnea

*** Normal temperature range^19^:

- Axillary: 34.7 - 37.3°C
- Oral: 35.5 - 37.5°C
- Rectal: 36.6 - 38.0°C

**References**

- - - 1. Mlinaric-Galinovic G, Varda-Brkic D. Nosocomial respiratory syncytial virus infection in children’s wards. Diag Micro Inf Dis 37(2000) 237-246.
      2. Ralston S, Hill V. Incidence of Apnea in Infants Hospitalized with RSV Bronchiolitis: A Systematic Review. The Journal of Pediatrics. 2009. 10:106
      3. Sazawal S et al. Effect of pneumonia case management on mortality in neonates, infants, and preschool children: a meta-analysis of community based trials. Lancet Infect Disease 2003; 3: 547-556.
      4. Shann F et al. Acute lower respiratory tract infections in children: possible criteria for selection of patients for antibiotic therapy and hospital admission. Bulletin of the World Health Organization. 1984. 62: 749-753.
      5. Cherian T, Mulholland EK, Carlin JB et al. Standardized interpretation of paediatric chest radiographs for the diagnosis of pneumonia in epidemiological studies. Bull World Health Organ. 2005; 83: 353-359.
      6. Margolis P, Gadomski A. The rationale clinical exam: does this infant have pneumonia? JAMA 1998: 279-308-313. Mahabee-Gittens EM et al. Identifying children with pneumonia in the emergency department. Clin Pediatr. 2005; 44:427-435.
      7. Cherian T, John TJ et al. Evaluation of clinical signs for the diagnosis of acute lower respiratory tract infection Lancet 1988. 2:125-8.
      8. Mulholland EK, Simoes EAF. Standardized diagnosis of pneumonia in developing countries. Pediatric infectious disease journal. 1992; 11: 77-81
      9. Gove S, for the WHO Working Group on Guidelines for Integrated Management of the sick child. Integrated management of childhood illness by outpatient health workers: technical basis and overview. Bulletin of the World Health Organization 1997; 75 Supp 1:7-16
      10. Horan T et al. CDC/NHSN surveillance definition of health care-associated infection and criteria for specific types of infections in the acute care setting. Am J Infect Control 2008;36:309-32.
      11. Subcommittee on Diagnosis and Management of Bronchiolitis. Diagnosis and Management of Bronchiolitis. Pediatrics 118;1774-1793. 2006
      12. McNally D, Leis K, Matheson L, Karuananyake c, Sankaran K, Rosenberg A. Vitamin D Deficiency in Young Children with Severe Acute Lower Respiratory Infection. Pediatrc Pulmonology. 44:981-988 2009
      13. Green G, Hood K, Little P, Verheij T, Goosens H, Coenen S, Butler C. Towards Clinical Definitions of lower respiratory tract infection (LRTI) for research and primary care practice in Europe: an international consensus study. Primary Care Respiratory Journal 20; 2011
      14. Jadavji T, Law B, Label M, Kennedy W, Gold R, Wang E. A practical guide or the diagnosis and treatment of pediatric pneumina. Can Med Assoc. 156(5);703-711. 1997
      15. Court SD. The definition of acute respiratory illnesses in children. Postgrad Med J 1973;49:771-6
      16. Skjerven H et al. Racemic Adrenaline and inhalation strategies in Acute bronchilitis. NEJM. 384 (24); 2286 – 2293. 2012
      17. Scott A et al. The definition of pneumonia, the assessment of severity and clinical standardiation in pneumina etiology research for child health study. Clin Inf Ds. 2012 54;109-16
      18. Bradley J et al. the Management of community-acquired pneumonia and infants and childrenolder than 3 months of age: clinical practice guidelines by the pediatric infectious disease society and the infectious disease society of America. Clin Inf Ds. 53 (7) 25-76. 2011
      19. Leduc D, Woods S; Canadian pediatric society, Community Paediatrics Committee. Temperature measurement in paediatrics. 2015. Available online:http://www.cps.ca/documents/position/temperature-measurement

**Table A. Algorithm validation by age at hospitalization, against the Canadian Institute of Health Information – Discharge Abstract Database.**

|  | Under 6 months | Over 6 month |
| --- | --- | --- |
| Sensitivity  (95% CI) | 97.5  (93.6, 99.3) | 98.5  (94.6, 99.8) |
| Specificity  (95% CI) | 99.7  (99.1, 99.9) | 99.4  (98.6, 99.8) |
| Positive predictive value  (95% CI) | 97.5  (93.6, 99.3) | 96.3  (91.6, 98.8) |
| Negative predictive value  (95% CI) | 99.7  (99.1, 99.9) | 99.8  (99.1, 100) |

Abbreviations: CI = Confidence Interval.

Algorithm used was any of the following: J12.1, J20.5, J21.0, and B97.4.

*Exact 95% CIs were calculated using the binomial distribution.

**Table B. Validation of HRU codes.**

|  | Non-invasive Ventilation | ICU  Admission | Endotracheal Intubation |
| --- | --- | --- | --- |
| Sensitivity  (95% CI) | 28.0  (12.1, 49.4) | 100  (93.5,100) | 94.4  (72.7,100) |
| Specificity  (95% CI) | 96.4  (81.7, 99.9) | 99.6  (98.7,100) | 100  (90.0,100) |
| Positive predictive value  (95% CI) | 87.5  (47.4, 99.7) | 96.5  (88.0,99.6) | 100  (80.5,100) |
| Negative predictive value  (95% CI) | 60.0  (44.3, 74.3) | 100  (99.3,100) | 97.2  (85.5,100) |

Abbreviations: CI = Confidence Interval; ICU = Intensive Care Unit.

*Exact 95% CIs were calculated using the binomial distribution.

**Table C. RSV admissions stratified by age between 2005-2013.**

| Year | Age < 1 | 1 ≤ Age < 3 | Age < 3 |
| --- | --- | --- | --- |
| 2005 | 6.28 (5.85,6.71) | 0.87 (0.76,0.99) | 2.72 (2.56,2.89) |
| 2006 | 11.98 (11.39,12.58) | 1.58 (1.43,1.74) | 5.12 (4.90,5.35) |
| 2007 | 9.35 (8.85,9.87) | 1.21 (1.08,1.35) | 4.01 (3.82,4.21) |
| 2008 | 7.49 (7.03,7.95) | 1.12 (0.99,1.25) | 3.30 (3.12,3.48) |
| 2009 | 10.2 (9.66,10.75) | 1.99 (1.83,2.17) | 4.71 (4.50,4.93) |
| 2010 | 7.59 (7.12,8.08) | 1.26 (1.13,1.40) | 3.33 (3.15,3.52) |
| 2011 | 11.99 (11.41,12.60) | 2.10 (1.93,2.29) | 5.48 (5.52,5.72) |
| 2012 | 10.18 (9.64,10.72) | 1.95 (1.78,2.13) | 4.76 (4.55,4.98) |
| 2013 | 9.78 (9.23,10.33) | 1.57 (1.43,1.73) | 4.31 (4.10,4.51) |

Data is presented as hospitalized RSV cases per 1000-person years. 95^th^ percentile confidence intervals provided in brackets and calculated using the gamma distribution.

Abbreviations: RSV = Respiratory Syncytial Virus.

**Table D. Standardized RSV hospital admissions by sex and age between 2005-2013.**

| Males | | | |
| --- | --- | --- | --- |
| Year | Age < 1 | 1 ≤ Age < 3 | Age < 3 |
| 2005 | 7.3 (6.67, 7.97) | 0.94 (0.78, 1.12) | 3.11 (2.87, 3.37) |
| 2006 | 13.94 (13.07, 14.86) | 1.86 (1.64, 2.11) | 5.96 (5.63, 6.31) |
| 2007 | 10.54 (9.79, 11.33) | 1.38 (1.18, 1.59) | 4.52 (4.23, 4.82) |
| 2008 | 8.79 (8.11, 9.51) | 1.13 (0.96, 1.32) | 3.75 (3.49, 4.03) |
| 2009 | 11.02 (10.25, 11.84) | 2.19 (1.95, 2.46) | 5.12 (4.82, 5.44) |
| 2010 | 8.61 (7.91, 9.35) | 1.42 (1.23, 1.64) | 3.76 (3.5, 4.04) |
| 2011 | 13.19 (12.35, 14.08) | 2.15 (1.91, 2.42) | 5.92 (5.59, 6.26) |
| 2012 | 11.78 (10.99, 12.62) | 2.07 (1.84, 2.33) | 5.4 (5.08, 5.73) |
| 2013 | 11.21 (10.43, 12.03) | 1.65 (1.44, 1.88) | 4.83 (4.54, 5.15) |

| Females | | | |
| --- | --- | --- | --- |
| Year | Age < 1 | 1 ≤ Age < 3 | Age < 3 |
| 2005 | 5.2 (4.66, 5.79) | 0.8 (0.65, 0.97) | 2.3 (2.09, 2.53) |
| 2006 | 9.91 (9.16, 10.71) | 1.28 (1.09, 1.5) | 4.21 (3.93, 4.52) |
| 2007 | 8.1 (7.43, 8.81) | 1.04 (0.87, 1.23) | 3.47 (3.21, 3.74) |
| 2008 | 6.12 (5.54, 6.74) | 1.11 (0.93, 1.3) | 2.82 (2.59, 3.07) |
| 2009 | 9.33 (8.6, 10.1) | 1.78 (1.56, 2.03) | 4.28 (3.99, 4.58) |
| 2010 | 6.53 (5.91, 7.19) | 1.09 (0.92, 1.29) | 2.85 (2.62, 3.1) |
| 2011 | 10.74 (9.96, 11.56) | 2.06 (1.82, 2.33) | 5.02 (4.71, 5.35) |
| 2012 | 8.46 (7.77, 9.2) | 1.82 (1.6, 2.07) | 4.09 (3.81, 4.39) |
| 2013 | 8.24 (7.55, 8.97) | 1.49 (1.29, 1.72) | 3.74 (3.47, 4.02) |

Data is presented as hospitalized RSV cases per 1000-person years. 95^th^ percentile confidence intervals provided in brackets and calculated using the gamma distribution.

Abbreviations: RSV = Respiratory Syncytial Virus.

**Figure A. Proportion of children hospitalized with RSV in whom major risk factors were present from 2005-2013.**


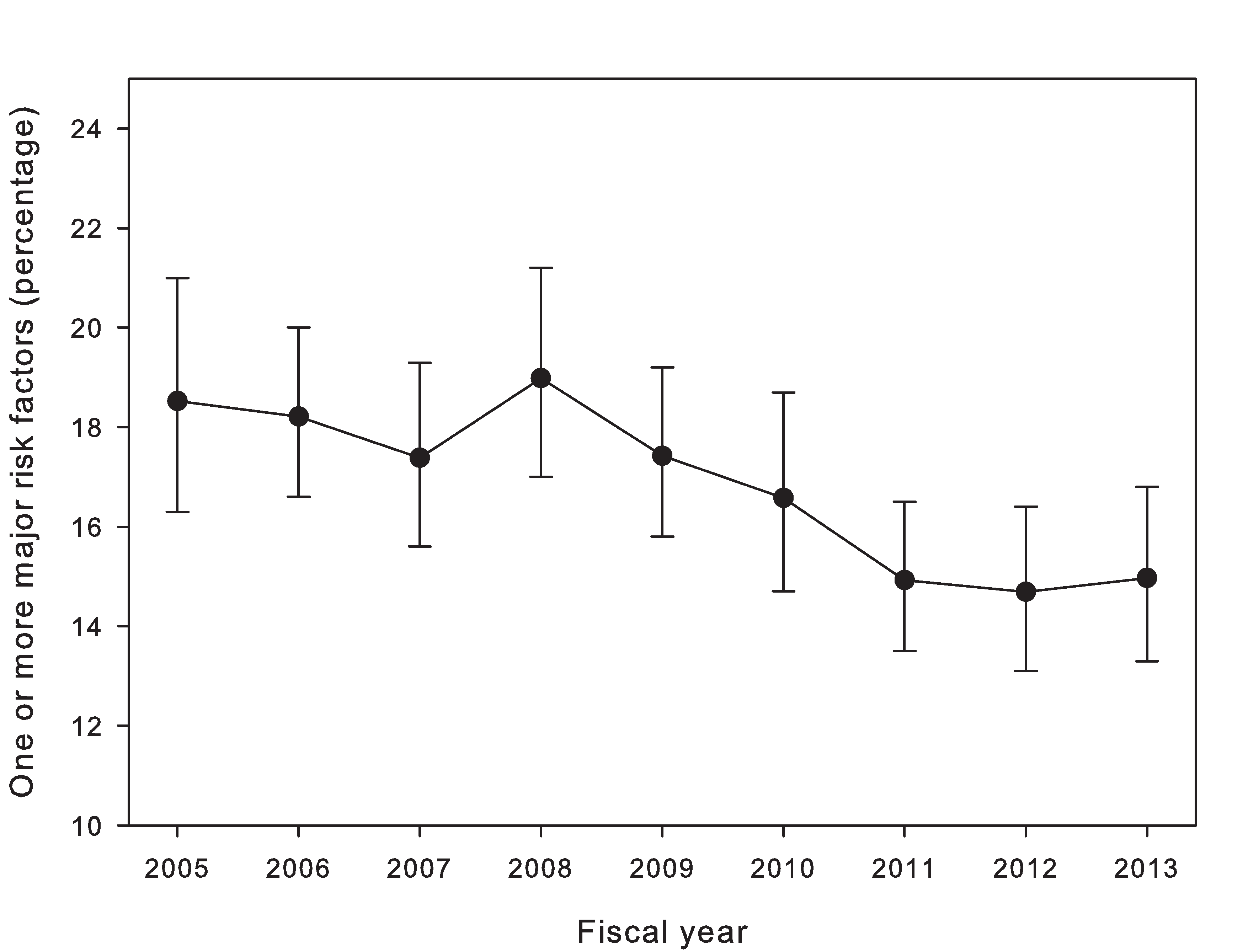


Legend: Change in major risk factors percentage over time. Shows the percentage of hospitalized RSV cases with one or more major risk factors by Fiscal Year. The error bars represent calculated 95% confidence intervals. A statistically significant change was calculated (-0.29 % per year, 95% CI: -0.05 to -0.53%, p=0.02).

Abbreviations: RSV = Respiratory Syncytial Virus.

**Figure B. PICU admission and intubation rates for children hospitalized with RSV 2005-2013.**


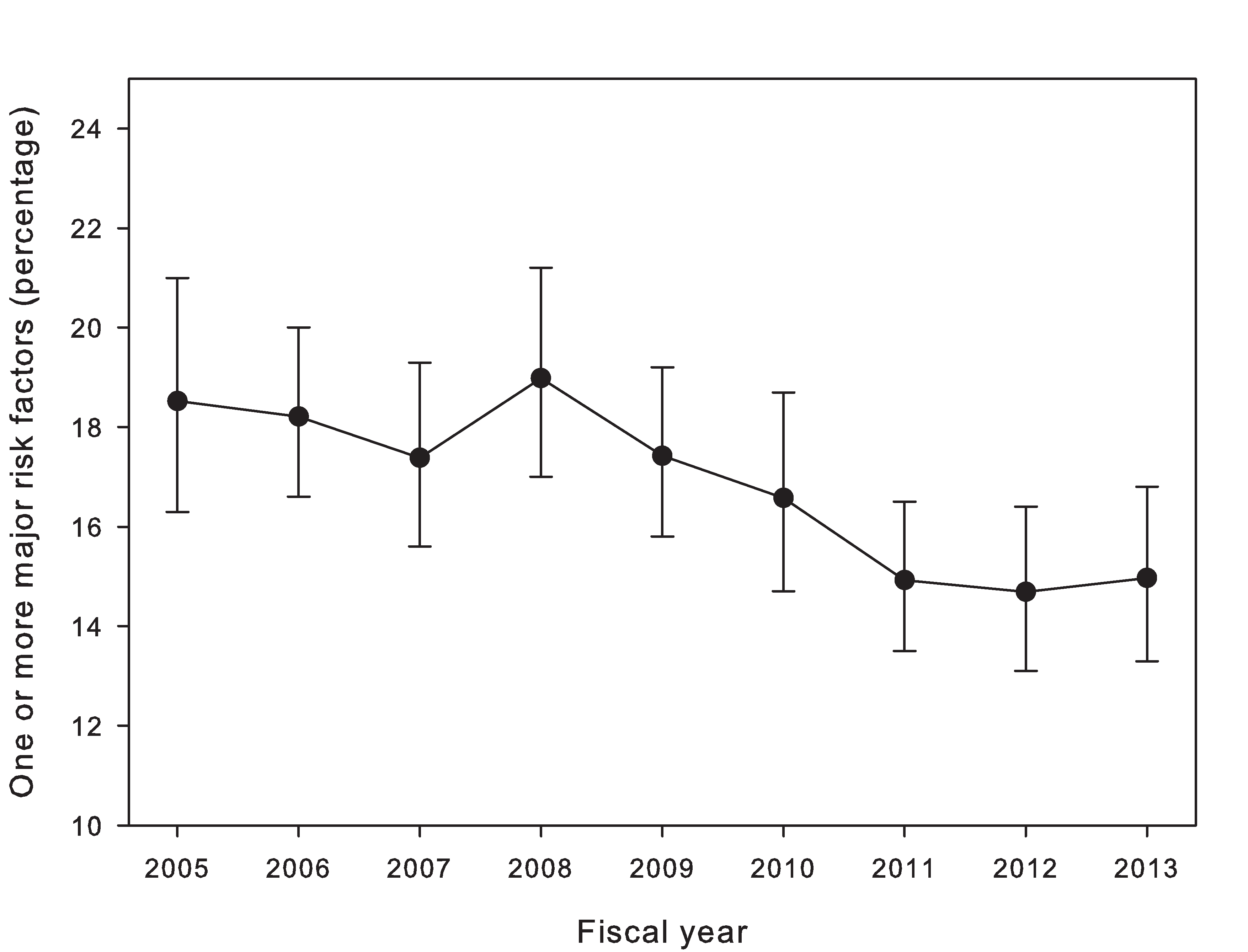

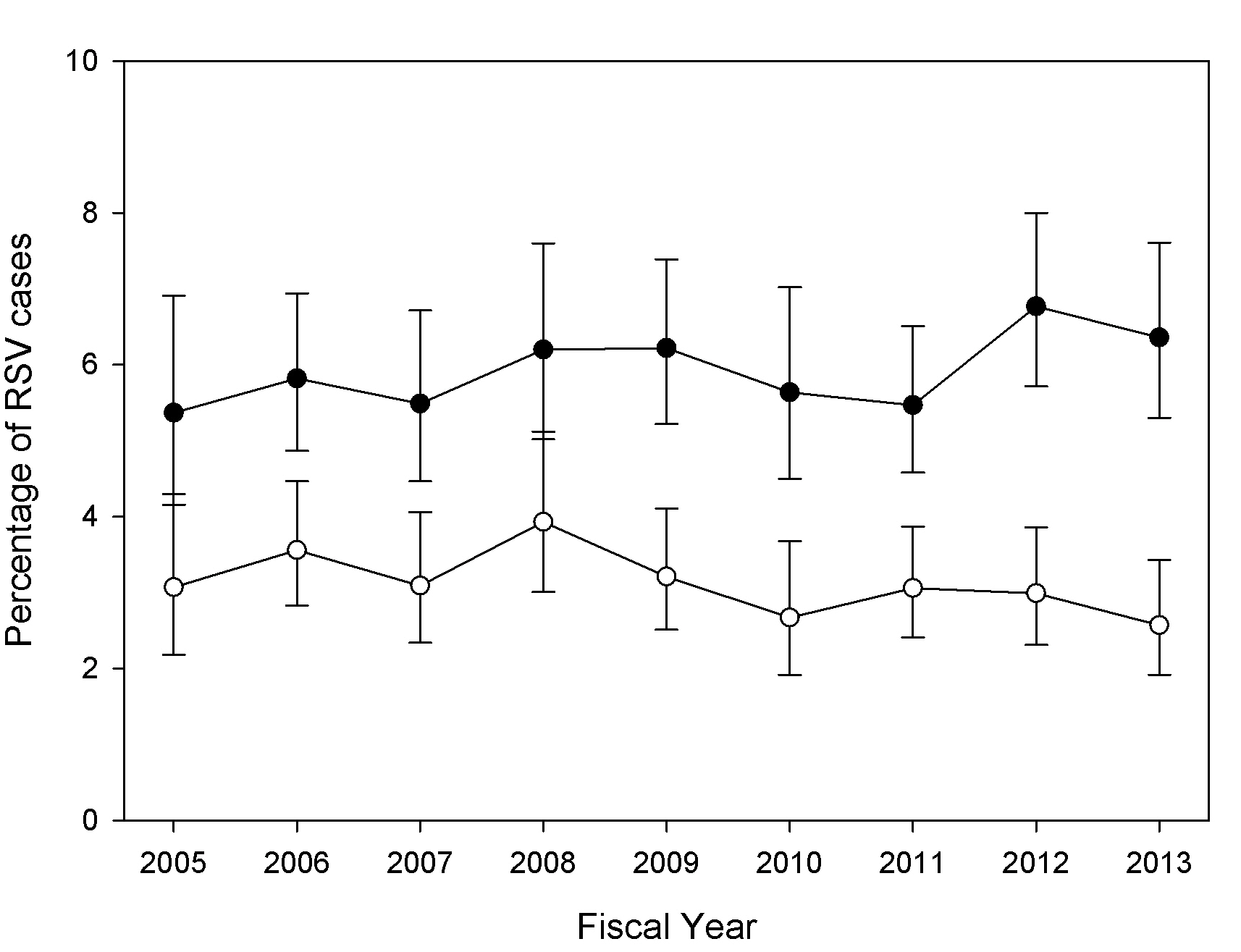


Legend: The percentage of hospitalized RSV cases that were admitted to PICU and intubated are shown. The error bars represent calculated 95% confidence intervals. Closed circles (•) represents the percentage of patients admitted to PICU each fiscal year. The open circles (ο) represents the percentage of hospitalized RSV cases that required intubation.

Abbreviations: RSV = Respiratory Syncytial Virus; PICU = Pediatric Intensive Care Unit.
