# Supplementary material for: Incidence of Hospitalization for Respiratory Syncytial Virus Infection amongst Children in Ontario, Canada: A Population-Based Study Using Validated Health Administrative Data
Source: PLoS One. 2016 Mar 9;11(3):e0150416. doi: 10.1371/journal.pone.0150416 (PMC4784925; doi:10.1371/journal.pone.0150416)
Supplement: S2 File — (DOCX) [file pone.0150416.s002.docx]

**Supporting Information**

**S2 File: Reporting Checklists**

**Checklist of reporting criteria for studies validating health administrative data algorithms.**

P = paragraph number. L = line number.

|  | YES | NO | UNCERTAIN | NOT  APPLICABLE | LOCATION |
| --- | --- | --- | --- | --- | --- |
| **TITLE, KEYWORDS, ABSTRACT:** | | | | | |
| Identify article as study of assessing diagnostic accuracy | √ |  |  |  | Abstract: study design, L3-6 |
| Identify article as study of administrative data | √ |  |  |  | Abstract: conclusion, L1-2 |
| **INTRODUCTION:** | | | | | |
| State disease identification & validation goals of study | √ |  |  |  | Intro: P3, L2-4 |
| **METHODS:** | | | | | |
| *Participants in validation cohort:* | | | | | |
| Describe validation cohort (Cohort of patients to which reference standard was applied): |  | | | | |
| • Age | √ |  |  |  | Methods P3 L1 |
| • Disease | √ |  |  |  | Methods P3 L25-17, Appendix A |
| • Severity | √ |  |  |  | Methods P4 L7-9 |
| • Location/Jurisdiction | √ |  |  |  | Methods P2 L1-2 and P3 L1-5, Appendix C |
| Describe recruitment procedure of validation cohort: |  | | | | |
| • Inclusion criteria | √ |  |  |  | Methods P3 L17-23, Appendix A |
| • Exclusion criteria | √ |  |  |  | Methods P3 L17-23, Appendix A |
| • Describe patient sampling (random, consecutive, all, etc.) | √ |  |  |  | Methods P3 L5-10 |
| Describe data collection |  | | | | |
| • Who identified patients and did selection adhere to patient recruitment criteria | √ |  |  |  | Methods P3, L1-32 |
| • Who collected data | √ |  |  |  | Methods P2 L31-34 |
| • A priori data collection form | √ |  |  |  | Methods P3 L28-29 |
| • Disease classification | √ |  |  |  | Methods P3 L25-28, Table 1, Appendix C |
| • Split sample (i.e. re-validation using a separate cohort) |  | √ |  |  | NA |
| *Test Methods:* | | | | | |
| Describe number, training and expertise of persons reading reference standard | √ |  |  |  | Methods P3 L29-32 |
| If >1 person reading reference standard, quote measure of consistency (e.g. kappa) |  | √ |  |  | NA |
| Blinding of interpreters of reference standard to results of classification by administrative data | √ |  |  |  | Methods P7 L1-3 |
| *Statistical Methods:* | | | | | |
| Describe methods of calculating/comparing diagnostic accuracy | √ |  |  |  | Methods P7 L1-5, P8 L1-9 |
| **RESULTS:** | | | | | |
| *Participants:* | | | | | |
| Report when study done, start/end dates of enrolment | √ |  |  |  | Methods P2 L1-3 |
| Describe number of people who satisfied inclusion/exclusion criteria | √ |  |  |  | Result: P1 L1-5 |
| Study flow diagram | √ |  |  |  | Figure 1 |
| *Test results:* | | | | | |
| Report distribution of disease severity | √ |  |  |  | Result: P1 L4-5, P2 L1-8 |
| Report cross-tabulation of index tests by results of reference standard |  | √ |  |  | It was not possible to provide the 2 x 2 table because some cell sizes were smaller than 6 and could not be reported, as per ICES privacy regulations |
| *Estimates:* | | | | | |
| Report at least 4 estimates of diagnostic accuracy | √ |  |  |  | Methods P4, L1-3 P6 L6-9 and Table 2 |
| Diagnostic Accuracy Measures Reported: |  | | | | |
| • Sensitivity | √ |  |  |  | Table 2 |
| • Specificity | √ |  |  |  | Table 2 |
| • PPV | √ |  |  |  | Table 2 |
| • NPV | √ |  |  |  | Table 2 |
| • Likelihood ratios |  | √ |  |  | NA |
| • Kappa |  | √ |  |  | Not complete |
| • Area under the ROC curve / c-statistic |  | √ |  |  | NA |
| • Accuracy/agreement |  | √ |  |  | NA |
| • Other (specify) |  | √ |  |  | NA |
| Report accuracy for subgroups (e.g. age, geography, different sex, etc.) | √ |  |  |  | Table 2, Results P |
| If PPV/NPV reported, ratio of cases/controls of validation cohort approximate prevalence of condition in the population | √ |  |  |  | Algorithm validation: P1, L1-8  Table 2 |
| Report 95% confidence intervals for each diagnostic measure | √ |  |  |  | Table 2 |
| **DISCUSSION:** | | | | | |
| Discuss the applicability of the validation findings | √ |  |  |  | Discussion: P1 L1-6, P2 L8-12 |

|  |  | STROBE items | RECORD items |
| --- | --- | --- | --- |
| Title and abstract | | | |
|  | 1 | (a) Indicate the study’s design with a commonly used term in the title or the abstract  **Complete: Title and Abstract P2 and P3**  (b) Provide in the abstract an informative and balanced summary of what was done and what was found  **Complete: Abstract P3 and P4** | 1.1: The type of data used should be specified in the title or abstract. When possible, the name of the databases used should be included.  **Complete: Abstract P2, L5-6 and P3 L5-6**  1.2: If applicable, the geographic region and timeframe within which the study took place should be reported in the title or abstract.  **Complete: Abstract P3, L1-3**  1.3: If linkage between databases was conducted for the study, this should be clearly stated in the title or abstract.  **Complete: Abstract P4. L2-3** |
| Introduction | | | |
| Background rationale | 2 | Explain the scientific background and rationale for the investigation being reported  **Complete: Introduction P1 and P2** |  |
| Objectives | 3 | State specific objectives, including any pre-specified hypotheses  **Complete: Introduction P3** |  |
| Methods | | | |
| Study Design | 4 | Present key elements of study design early in the paper  **Complete: Methods P2 and P3** |  |
| Setting | 5 | Describe the setting, locations, and relevant dates, including periods of recruitment, exposure, follow-up, and data collection  **Complete: Methods P3 and P4, Appendix A, Appendix B** |  |
| Participants | 6 | (a)Cohort study - Give the eligibility criteria, and the sources and methods of selection of participants. Describe methods of follow- up Case-control study - Give the eligibility criteria, and the sources and methods of case ascertainment and control selection. Give the rationale for the choice of cases and controls  Cross-sectional study - Give the eligibility criteria, and the sources and methods of selection of participants  **Complete: Appendix A, Appendix B and Methods P3, L6-10**  (b) Cohort study - For matched studies, give matching criteria and number of exposed and unexposed  Case-control study - For matched studies, give matching criteria and the number of controls per case  **NA** | 6.1: The methods of study population selection (such as codes or algorithms used to identify subjects) should be listed in detail. If this is not possible, an explanation should be provided.  **Complete: Appendix A, Appendix B and Methods P3, L6-10**  6.2: Any validation studies of the codes or algorithms used to select the population should be referenced. If validation was conducted for this study and not published elsewhere, detailed methods and results should be provided.  **Complete: Table 1 and Table 2 (algorithm used for cohort creation)**  6.3: If the study involved linkage of databases, consider use of a flow diagram or other graphical display to demonstrate the data linkage process, including the number of individuals with linked data at each stage.  **Complete: Figure 1, Methods P2 L6 and Results P2 L1-3** |
| Variables | 7 | Clearly define all outcomes, exposures, predictors, potential confounders, and effect modifiers. Give diagnostic criteria, if applicable.  **Complete: Table 1, Table 3** | 7.1: A complete list of codes and algorithms used to classify exposures, outcomes, confounders, and effect modifiers should be provided. If these cannot be reported, an explanation should be provided.  **Compete: Table 1 and Appendix A** |
| Data sources and measurement | 8 | For each variable of interest, give sources of data and details of methods of assessment (measurement). Describe comparability of assessment methods if there is more than one group  **Complete: Appendix A, Table 1** |  |
| Bias |  | Describe any efforts to address potential sources of bias  **Not complete/Not relevant** |  |
| Study size | 10 | Explain how the study size was arrived at  **Complete: Methods - P5 L4-9** |  |
| Quantitative variables |  | Explain how quantitative variables were handled in the analyses. If applicable, describe which groupings were chosen, and why  Not relevant. We dealt with quantitative variables by saying that what was determined from the chart review had to match exactly with CIHI-DAD |  |
| Statistical Analyses | 12 | (a) Describe all statistical methods, including those used to control for confounding  **Complete: Methods P7 and P8**  (b) Describe any methods used to examine subgroups and interactions  **Complete: Methods P5 L4-9**  (c) Explain how missing data were addressed  **Complete: Results P2 L1-4; P3 L 3-6**  (d) Cohort study - If applicable, explain how loss to follow-up was addressed Case-control study - If applicable, explain how matching of cases and controls was addressed Cross-sectional study - If applicable, describe analytical methods taking account of sampling strategy  **NA**  (e) Describe any sensitivity analyses  **Complete: Methods P7** |  |
| Data Access and Cleaning Methods |  |  | 12.1: Authors should describe the extent to which the investigators had access to the database population used to create the study population.  **Not complete**  12.2: Authors should provide information on the data cleaning methods used in the study.  **Not complete**  12.3: State whether the study included person- level, institutional-level, or other data linkage across two or more databases. The methods of linkage and methods of linkage quality evaluation should be provided.  **Complete: Methods P2 L6-7, Results P2** |
| Linkage |  |  |  |
| Results | | | |
| Participants | 13 | (a) Report the numbers of individuals at each stage of the study (e.g., numbers potentially eligible, examined for eligibility, confirmed eligible, included in the study, completing follow-up, and analysed)  **Complete: Results P1 and P2**  (b) Give reasons for non- participation at each stage.  **NA**  (c) Consider use of a flow diagram  **Complete: Figure 1** | 13.1: Describe in detail the selection of the persons included in the study (i.e., study population selection) including filtering based on data quality, data availability and linkage. The selection of included persons can be described in the text and/or by means of the study flow diagram.  **Complete: Methods P2, Methods P3, Figure 1, Appendix A and B, Results P2** |
| Descriptive data | 14 | (a) Give characteristics of study participants (e.g., demographic, clinical, social) and information on exposures and potential confounders  **Complete: Results P1, Appendix C**  (b) Indicate the number of participants with missing data for each variable of interest  **Complete: Figure 1 and Results P2**  (c) Cohort study - summarise follow-up time (e.g., average and total amount)  **NA** |  |
| Outcome data | 15 | Cohort study - Report numbers of outcome events or summary measures over time Case-control study - Report numbers in each exposure category, or summary measures of exposure  Cross-sectional study - Report numbers of outcome events or summary measures  **Complete: Table 2, Results P3 and Results P4** |  |
| Main results | 16 | (a) Give unadjusted estimates and, if applicable, confounder-adjusted estimates and their precision (e.g., 95% confidence interval). Make clear which confounders were adjusted for and why they were included  **Complete: Table 2, Results P3**  (b) Report category boundaries when continuous variables were categorized  **Only applicable for subgroup analysis of risk factors.**  **Complete: Table 3**  (c) If relevant, consider translating estimates of relative risk into absolute risk for a meaningful time period  **NA** |  |
| Other analyses | 17 | Report other analyses done— e.g., analyses of subgroups and interactions, and sensitivity analyses  **Complete: Subgroup - Results P4; Incidence - Results P6; Risk factors - Results P7** |  |
| Discussion | | | |
| Key results | 18 | Summarise key results with reference to study objectives  **Complete: Results P1** | 19.1: Discuss the implications of using data that were not created or collected to answer the specific research question(s). Include discussion of misclassification bias, unmeasured confounding, missing data, and changing eligibility over time, as they pertain to the study being reported.  **Complete: Results P2 L9-12 and L22-24 and P5** |
| Limitations | 19 | Discuss limitations of the study, taking into account sources of potential bias or imprecision. Discuss both direction and magnitude of any potential bias  **Complete: Results P2 L9-12 and L22-24, P5** |  |
| Interpretation | 20 | Give a cautious overall interpretation of results considering objectives, limitations, multiplicity of analyses, results from similar studies, and other relevant evidence  **Complete: Results P2 L9-12 and L22-24** |  |
| Generalisability | 21 | Discuss the generalisability (external validity) of the study results  **Complete: Results P2 and P3** |  |
| Other Information | | | |
| Funding | 22 | Give the source of funding and the role of the funders for the present study and, if applicable, for the original study on which the present article is based  **Complete: Acknowledgement Section P1** |  |
|  |  |  | 22.1: Authors should provide information on how to access any supplemental information such as the study protocol, raw data, or programming code.  **Not applicable** |
